# (*E*)-1-[1-(2-Chloro­phen­yl)ethyl­idene]-2-(2,4-dinitro­phen­yl)hydrazine

**DOI:** 10.1107/S160053681105001X

**Published:** 2011-11-30

**Authors:** Suchada Chantrapromma, Boonlerd Nilwanna, Patcharaporn Jansrisewangwong, Thawanrat Kobkeatthawin, Hoong-Kun Fun

**Affiliations:** a Crystal Materials Research Unit, Department of Chemistry, Faculty of Science, Prince of Songkla University, Hat-Yai, Songkhla 90112, Thailand; b X-ray Crystallography Unit, School of Physics, Universiti Sains Malaysia, 11800 USM, Penang, Malaysia

## Abstract

The title mol­ecule, C_14_H_11_ClN_4_O_4_, is in an *E* configuration and is twisted with the dihedral angle between the two benzene rings being 38.48 (8)°. The ethyl­idenehydrazine plane makes dihedral angles of 6.03 (10) and 44.04 (11)°, respectively, with the dinitro- and chloro-substituted benzene rings. The two nitro groups are essentially coplanar with the bound benzene ring, making dihedral angles of 0.9 (2) and 1.65 (18)°. An intra­molecular N—H⋯O hydrogen bond generates an *S*(6) ring motif. In the crystal, mol­ecules are linked by a weak C—H⋯O inter­action into a chain along the *c* axis. The chains are further stacked along the *b* axis by a π–π inter­action with a centroid–centroid distance of 3.6088 (10) Å.

## Related literature

For bond-length data, see: Allen *et al.* (1987 ▶). For hydrogen-bond motifs, see: Bernstein *et al.* (1995 ▶). For related structures, see: Fun *et al.* (2010 ▶, 2011 ▶); Jansrisewangwong *et al.* (2010 ▶); Nilwanna *et al.* (2011 ▶). For background to and the biological activity of hydro­zones, see: Angelusiu *et al.* (2010 ▶); Bendre *et al.* (1998 ▶); Gokce *et al.* (2009 ▶); Li *et al.* (2008 ▶); Loncle *et al.* (2004 ▶).
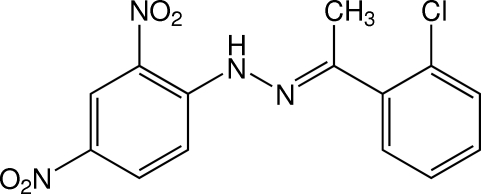


## Experimental

### 

#### Crystal data

C_14_H_11_ClN_4_O_4_
*M* *_r_* = 334.72
Monoclinic, 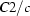

*a* = 32.660 (3) Å
*b* = 7.1435 (7) Å
*c* = 13.4798 (13) Å
β = 112.215 (2)°
*V* = 2911.5 (5) Å^3^
*Z* = 8
Mo *K*α radiation
μ = 0.29 mm^−1^
*T* = 297 K
0.36 × 0.26 × 0.15 mm

#### Data collection

Bruker APEXII CCD area-detector diffractometer
Absorption correction: multi-scan (*SADABS*; Bruker, 2009 ▶) *T*_min_ = 0.904, *T*_max_ = 0.957
16146 measured reflections
4458 independent reflections
3036 reflections with *I* > 2σ(*I*)
*R*_int_ = 0.028

#### Refinement

*R*[*F* ^2^ > 2σ(*F* ^2^)] = 0.042
*wR*(*F* ^2^) = 0.127
*S* = 1.04
4458 reflections
213 parameters
H atoms treated by a mixture of independent and constrained refinement
Δρ_max_ = 0.24 e Å^−3^
Δρ_min_ = −0.27 e Å^−3^

### 

Data collection: *APEX2* (Bruker, 2009 ▶); cell refinement: *SAINT* (Bruker, 2009 ▶); data reduction: *SAINT*; program(s) used to solve structure: *SHELXTL* (Sheldrick, 2008 ▶); program(s) used to refine structure: *SHELXTL*; molecular graphics: *SHELXTL*; software used to prepare material for publication: *SHELXTL* and *PLATON* (Spek, 2009 ▶).

## Supplementary Material

Crystal structure: contains datablock(s) global, I. DOI: 10.1107/S160053681105001X/is5008sup1.cif

Structure factors: contains datablock(s) I. DOI: 10.1107/S160053681105001X/is5008Isup2.hkl

Supplementary material file. DOI: 10.1107/S160053681105001X/is5008Isup3.cml

Additional supplementary materials:  crystallographic information; 3D view; checkCIF report

## Figures and Tables

**Table 1:** Hydrogen-bond geometry (Å, °)

*D*—H⋯*A*	*D*—H	H⋯*A*	*D*⋯*A*	*D*—H⋯*A*
N2—H1*N*1⋯O1	0.85 (2)	1.97 (2)	2.6081 (19)	131.2 (17)
C6—H6*A*⋯O3^i^	0.93	2.52	3.251 (2)	135

Symmetry code: (i) 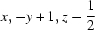.

**Fig. 1.**
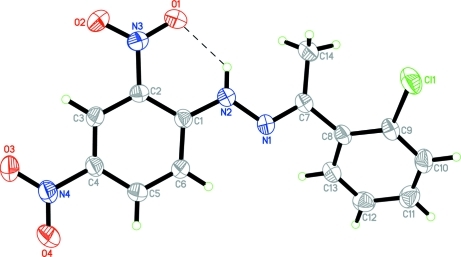
The molecular structure of the title compound, showing 40% probability displacement ellipsoids and the atom-numbering scheme. Hydrogen bond is shown as a dashed line.

**Fig. 2.**
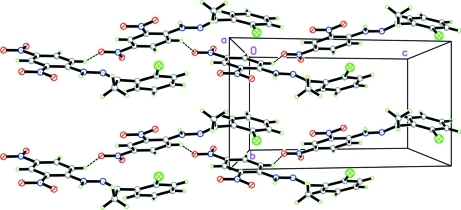
A crystal packing diagram of the title compound viewed along the a axis, showing chains running along the c axis. Hydrogen bonds are shown as dashed lines.

## supplementary crystallographic information

### Comment

Hydrazones have been known to be responsible for various bioactivities such as antibacterial (Angelusiu *et al.*, 2010), antioxidant (Li *et al.*, 2008), antifungal (Loncle *et al.*, 2004), anti-inflammatory (Gokce *et al.*, 2009) and also tyrosinase inhibitory (Bendre *et al.*, 1998) activities. With our on-going research on medicinal chemistry, we previously reported the syntheses and crystal structures of some hydrazone derivatives (Fun *et al.*, 2010, 2011; Jansrisewangwong *et al.*, 2010; Nilwanna *et al.*, 2011). Herein we report the crystal structure of the title compound. It was screened for antioxidant and antibacterial activities and found to be inactive.

The title molecule (Fig. 1), C_14_H_11_ClN_4_O_4_, is twisted and exists in an *E* configuration with respect to the ethylidene C7═N1 double bond [1.2877 (17) Å] with the torsion angle N2–N1–C7–C8 = -176.69 (13)°. The dihedral angle between the benzene rings of the 2,4-dinitrophenyl and 2-chlorophenyl groups is 38.48 (8)°. The middle ethylidenehydrazine unit (C7/C14/N1/N2) is planar with an *r.m.s* deviation of 0.0040 (1) Å and the torsion angle of N2–N1–C7–C14 is -1.3 (2)°. This middle C/C/N/N plane makes the dihedral angles of 6.03 (10) and 44.04 (11)° with the 2,4-dinitrophenyl and 2-chlorophenyl rings, respectively. The two nitro groups of 2,4-dinitrophenyl are essentially co-planar with the bound benzene ring with an *r.m.s*. deviation of 0.0081 (1) Å for the twelve non H-atoms and the O–N–C–C angles are -0.3 (2), 0.2 (2), 0.1 (3) and -0.1 (3)°. An intramolecular N—H···O hydrogen bond between the hydrazone-NH and the *ortho* nitro group (Fig. 1 and Table 1) generates an S(6) ring motif (Bernstein *et al.*, 1995). The bond distances are within the normal range (Allen *et al.*, 1987) and are comparable with related structures (Fun *et al.*, 2010, 2011; Jansrisewangwong *et al.*, 2010; Nilwanna *et al.*, 2011).

In the crystal structure (Fig. 2), the molecules are linked by weak C—H···O interactions (Table 1) into chains along the *c* axis in a head-to-head manner. These chains are further stacked along the *b* axis by a π–π interaction with *Cg*1···*Cg*2^ii^ distance of 3.6088 (10) Å [symmetry code: (ii) *x*, 1 - *y*, 1/2 + *z*]; *Cg*1 and *Cg*2 are the centroids of C1–C6 and C8–C13 benzene rings, respectively.

### Experimental

The title compound (I) was synthesized by dissolving 2,4-dinitrophenylhydrazine (0.40 g, 2 mmol) in ethanol (10.00 ml) and H_2_SO_4_ (conc.) (98%, 0.50 ml) was slowly added with stirring. 2-Chloroacetophenone (0.30 ml, 2 mmol) was then added to the solution with continuous stirring. The solution was refluxed for 1 h yielding a yellow solid, which was filtered off and washed with methanol. Yellow block-shaped single crystals of the title compound suitable for X-ray diffraction were recrystalized from ethanol by slow evaporation of the solvent at room temperature over several days (m.p. 478–479).

### Refinement

Amide H atom was located in a difference map and refined isotropically [N—H = 0.85 (2) Å]. The remaining H atoms were positioned geometrically and allowed to ride on their parent atoms, with C—H = 0.93 Å for aromatic and 0.96 Å for CH_3_ atoms. The *U*_iso_ values were constrained to be 1.5*U*_eq_ of the carrier atom for methyl H atoms and 1.2*U*_eq_ for the remaining H atoms. A rotating group model was used for the methyl groups.

### Crystal data

**Table:**

C_14_H_11_ClN_4_O_4_	*F*(000) = 1376
*M**_r_* = 334.72	*D*_x_ = 1.527 Mg m^−^^3^
Monoclinic, *C*2/*c*	Melting point = 478–479 K
Hall symbol: -C 2yc	Mo *K*α radiation, λ = 0.71073 Å
*a* = 32.660 (3) Å	Cell parameters from 4458 reflections
*b* = 7.1435 (7) Å	θ = 1.4–30.6°
*c* = 13.4798 (13) Å	µ = 0.29 mm^−^^1^
β = 112.215 (2)°	*T* = 297 K
*V* = 2911.5 (5) Å^3^	Block, yellow
*Z* = 8	0.36 × 0.26 × 0.15 mm

### Data collection

**Table:**

Bruker APEXII CCD area-detector diffractometer	4458 independent reflections
Radiation source: sealed tube	3036 reflections with *I* > 2σ(*I*)
graphite	*R*_int_ = 0.028
φ and ω scans	θ_max_ = 30.6°, θ_min_ = 1.4°
Absorption correction: multi-scan (*SADABS*; Bruker, 2009)	*h* = −46→46
*T*_min_ = 0.904, *T*_max_ = 0.957	*k* = −10→10
16146 measured reflections	*l* = −19→19

### Refinement

**Table:**

Refinement on *F*^2^	Primary atom site location: structure-invariant direct methods
Least-squares matrix: full	Secondary atom site location: difference Fourier map
*R*[*F*^2^ > 2σ(*F*^2^)] = 0.042	Hydrogen site location: inferred from neighbouring sites
*wR*(*F*^2^) = 0.127	H atoms treated by a mixture of independent and constrained refinement
*S* = 1.04	*w* = 1/[σ^2^(*F*_o_^2^) + (0.0537*P*)^2^ + 1.2234*P*] where *P* = (*F*_o_^2^ + 2*F*_c_^2^)/3
4458 reflections	(Δ/σ)_max_ = 0.001
213 parameters	Δρ_max_ = 0.24 e Å^−^^3^
0 restraints	Δρ_min_ = −0.27 e Å^−^^3^

### Special details

**Table:**

Geometry. All e.s.d.'s (except the e.s.d. in the dihedral angle between two l.s. planes) are estimated using the full covariance matrix. The cell e.s.d.'s are taken into account individually in the estimation of e.s.d.'s in distances, angles and torsion angles; correlations between e.s.d.'s in cell parameters are only used when they are defined by crystal symmetry. An approximate (isotropic) treatment of cell e.s.d.'s is used for estimating e.s.d.'s involving l.s. planes.
Refinement. Refinement of *F*^2^ against ALL reflections. The weighted *R*-factor *wR* and goodness of fit *S* are based on *F*^2^, conventional *R*-factors *R* are based on *F*, with *F* set to zero for negative *F*^2^. The threshold expression of *F*^2^ > σ(*F*^2^) is used only for calculating *R*-factors(gt) *etc*. and is not relevant to the choice of reflections for refinement. *R*-factors based on *F*^2^ are statistically about twice as large as those based on *F*, and *R*- factors based on ALL data will be even larger.

### Fractional atomic coordinates and isotropic or equivalent isotropic displacement parameters (Å^2^)

**Table:**

	*x*	*y*	*z*	*U*_iso_*/*U*_eq_	
Cl1	0.015926 (14)	0.34149 (7)	0.42093 (4)	0.06172 (16)	
O1	0.06808 (4)	0.2345 (2)	0.95156 (10)	0.0573 (3)	
O2	0.09965 (5)	0.2876 (2)	1.12043 (10)	0.0700 (4)	
O3	0.24764 (5)	0.5021 (3)	1.28114 (10)	0.0848 (5)	
O4	0.28607 (4)	0.5486 (3)	1.18505 (11)	0.0805 (5)	
N1	0.11688 (4)	0.28366 (18)	0.72895 (9)	0.0409 (3)	
N2	0.10981 (4)	0.2894 (2)	0.82297 (10)	0.0419 (3)	
H1N1	0.0863 (7)	0.249 (3)	0.8280 (15)	0.053 (5)*	
N3	0.10020 (4)	0.2853 (2)	1.03026 (11)	0.0449 (3)	
N4	0.25170 (5)	0.5037 (2)	1.19483 (11)	0.0556 (4)	
C1	0.14355 (5)	0.3406 (2)	0.91423 (11)	0.0360 (3)	
C2	0.14038 (4)	0.3427 (2)	1.01624 (11)	0.0367 (3)	
C3	0.17560 (5)	0.3958 (2)	1.10769 (11)	0.0408 (3)	
H3A	0.1729	0.3959	1.1739	0.049*	
C4	0.21443 (5)	0.4482 (2)	1.09923 (11)	0.0421 (3)	
C5	0.21904 (5)	0.4482 (2)	1.00072 (12)	0.0457 (4)	
H5A	0.2457	0.4844	0.9964	0.055*	
C6	0.18447 (5)	0.3951 (2)	0.91068 (11)	0.0433 (3)	
H6A	0.1879	0.3947	0.8453	0.052*	
C7	0.08535 (5)	0.2174 (2)	0.64630 (11)	0.0385 (3)	
C8	0.09681 (5)	0.2063 (2)	0.54960 (11)	0.0388 (3)	
C9	0.06866 (5)	0.2561 (2)	0.44575 (12)	0.0425 (3)	
C10	0.08208 (6)	0.2441 (3)	0.35979 (13)	0.0516 (4)	
H10A	0.0628	0.2781	0.2915	0.062*	
C11	0.12381 (7)	0.1822 (3)	0.37579 (14)	0.0565 (4)	
H11A	0.1329	0.1738	0.3183	0.068*	
C12	0.15241 (6)	0.1321 (3)	0.47736 (15)	0.0539 (4)	
H12A	0.1807	0.0900	0.4882	0.065*	
C13	0.13910 (5)	0.1445 (2)	0.56248 (13)	0.0456 (3)	
H13A	0.1588	0.1109	0.6304	0.055*	
C14	0.04246 (5)	0.1437 (3)	0.64700 (14)	0.0546 (4)	
H14A	0.0482	0.0642	0.7082	0.082*	
H14B	0.0276	0.0732	0.5827	0.082*	
H14C	0.0241	0.2465	0.6505	0.082*	

### Atomic displacement parameters (Å^2^)

**Table:**

	*U*^11^	*U*^22^	*U*^33^	*U*^12^	*U*^13^	*U*^23^
Cl1	0.0465 (2)	0.0735 (3)	0.0510 (2)	0.0134 (2)	0.00243 (17)	0.0014 (2)
O1	0.0378 (6)	0.0795 (9)	0.0541 (7)	−0.0125 (6)	0.0168 (5)	−0.0065 (6)
O2	0.0663 (8)	0.1052 (11)	0.0496 (7)	−0.0225 (8)	0.0345 (6)	−0.0097 (7)
O3	0.0585 (8)	0.1510 (16)	0.0417 (7)	−0.0191 (9)	0.0154 (6)	−0.0282 (8)
O4	0.0416 (7)	0.1350 (14)	0.0594 (8)	−0.0233 (8)	0.0127 (6)	−0.0197 (9)
N1	0.0388 (6)	0.0484 (7)	0.0323 (6)	−0.0033 (5)	0.0097 (5)	−0.0021 (5)
N2	0.0358 (6)	0.0539 (8)	0.0343 (6)	−0.0052 (6)	0.0114 (5)	−0.0027 (5)
N3	0.0421 (7)	0.0511 (8)	0.0458 (7)	−0.0034 (6)	0.0214 (6)	−0.0025 (6)
N4	0.0406 (7)	0.0801 (11)	0.0413 (7)	−0.0039 (7)	0.0099 (6)	−0.0137 (7)
C1	0.0336 (6)	0.0390 (7)	0.0335 (6)	0.0020 (5)	0.0106 (5)	0.0000 (5)
C2	0.0334 (6)	0.0403 (7)	0.0381 (7)	0.0004 (5)	0.0152 (5)	−0.0013 (5)
C3	0.0409 (7)	0.0491 (8)	0.0338 (6)	0.0015 (6)	0.0156 (6)	−0.0038 (6)
C4	0.0338 (7)	0.0522 (9)	0.0358 (7)	0.0005 (6)	0.0079 (5)	−0.0063 (6)
C5	0.0331 (7)	0.0616 (10)	0.0427 (8)	−0.0019 (7)	0.0147 (6)	−0.0037 (7)
C6	0.0367 (7)	0.0597 (10)	0.0345 (7)	−0.0025 (7)	0.0147 (6)	−0.0016 (6)
C7	0.0343 (7)	0.0400 (7)	0.0359 (7)	0.0005 (6)	0.0072 (5)	0.0002 (6)
C8	0.0371 (7)	0.0383 (7)	0.0345 (6)	−0.0045 (6)	0.0062 (5)	−0.0045 (5)
C9	0.0408 (7)	0.0411 (8)	0.0366 (7)	−0.0020 (6)	0.0046 (6)	−0.0049 (6)
C10	0.0599 (10)	0.0528 (10)	0.0349 (7)	−0.0056 (8)	0.0099 (7)	−0.0042 (6)
C11	0.0673 (11)	0.0608 (11)	0.0454 (9)	−0.0083 (9)	0.0257 (8)	−0.0083 (8)
C12	0.0476 (9)	0.0564 (10)	0.0600 (10)	−0.0022 (8)	0.0229 (8)	−0.0086 (8)
C13	0.0394 (7)	0.0494 (9)	0.0415 (7)	−0.0004 (6)	0.0080 (6)	−0.0041 (6)
C14	0.0411 (8)	0.0684 (12)	0.0477 (9)	−0.0113 (8)	0.0094 (7)	0.0021 (8)

### Geometric parameters (Å, °)

**Table:**

Cl1—C9	1.7364 (16)	C5—H5A	0.9300
O1—N3	1.2314 (17)	C6—H6A	0.9300
O2—N3	1.2224 (17)	C7—C8	1.488 (2)
O3—N4	1.2198 (19)	C7—C14	1.500 (2)
O4—N4	1.2213 (19)	C8—C13	1.397 (2)
N1—C7	1.2877 (17)	C8—C9	1.399 (2)
N1—N2	1.3720 (17)	C9—C10	1.388 (2)
N2—C1	1.3551 (18)	C10—C11	1.370 (3)
N2—H1N1	0.85 (2)	C10—H10A	0.9300
N3—C2	1.4537 (19)	C11—C12	1.381 (3)
N4—C4	1.4537 (19)	C11—H11A	0.9300
C1—C6	1.410 (2)	C12—C13	1.375 (2)
C1—C2	1.4176 (19)	C12—H12A	0.9300
C2—C3	1.384 (2)	C13—H13A	0.9300
C3—C4	1.368 (2)	C14—H14A	0.9600
C3—H3A	0.9300	C14—H14B	0.9600
C4—C5	1.392 (2)	C14—H14C	0.9600
C5—C6	1.362 (2)		
			
C7—N1—N2	116.82 (13)	N1—C7—C8	113.18 (13)
C1—N2—N1	118.92 (13)	N1—C7—C14	124.54 (14)
C1—N2—H1N1	118.0 (13)	C8—C7—C14	122.11 (13)
N1—N2—H1N1	122.6 (13)	C13—C8—C9	116.70 (14)
O2—N3—O1	122.17 (13)	C13—C8—C7	118.19 (13)
O2—N3—C2	118.61 (13)	C9—C8—C7	125.09 (14)
O1—N3—C2	119.21 (12)	C10—C9—C8	121.61 (15)
O3—N4—O4	122.76 (14)	C10—C9—Cl1	117.66 (12)
O3—N4—C4	119.10 (14)	C8—C9—Cl1	120.71 (12)
O4—N4—C4	118.13 (14)	C11—C10—C9	119.86 (15)
N2—C1—C6	119.97 (13)	C11—C10—H10A	120.1
N2—C1—C2	123.43 (13)	C9—C10—H10A	120.1
C6—C1—C2	116.60 (12)	C10—C11—C12	119.94 (16)
C3—C2—C1	121.74 (13)	C10—C11—H11A	120.0
C3—C2—N3	116.67 (12)	C12—C11—H11A	120.0
C1—C2—N3	121.58 (12)	C13—C12—C11	120.12 (16)
C4—C3—C2	119.05 (13)	C13—C12—H12A	119.9
C4—C3—H3A	120.5	C11—C12—H12A	119.9
C2—C3—H3A	120.5	C12—C13—C8	121.76 (15)
C3—C4—C5	121.16 (13)	C12—C13—H13A	119.1
C3—C4—N4	119.52 (13)	C8—C13—H13A	119.1
C5—C4—N4	119.31 (14)	C7—C14—H14A	109.5
C6—C5—C4	119.88 (14)	C7—C14—H14B	109.5
C6—C5—H5A	120.1	H14A—C14—H14B	109.5
C4—C5—H5A	120.1	C7—C14—H14C	109.5
C5—C6—C1	121.56 (13)	H14A—C14—H14C	109.5
C5—C6—H6A	119.2	H14B—C14—H14C	109.5
C1—C6—H6A	119.2		
			
C7—N1—N2—C1	173.61 (14)	C4—C5—C6—C1	0.4 (3)
N1—N2—C1—C6	2.9 (2)	N2—C1—C6—C5	179.70 (15)
N1—N2—C1—C2	−176.94 (14)	C2—C1—C6—C5	−0.5 (2)
N2—C1—C2—C3	179.97 (14)	N2—N1—C7—C8	−176.69 (13)
C6—C1—C2—C3	0.1 (2)	N2—N1—C7—C14	−1.3 (2)
N2—C1—C2—N3	1.3 (2)	N1—C7—C8—C13	41.4 (2)
C6—C1—C2—N3	−178.56 (14)	C14—C7—C8—C13	−134.06 (16)
O2—N3—C2—C3	0.2 (2)	N1—C7—C8—C9	−137.42 (16)
O1—N3—C2—C3	−179.10 (15)	C14—C7—C8—C9	47.1 (2)
O2—N3—C2—C1	179.01 (15)	C13—C8—C9—C10	0.2 (2)
O1—N3—C2—C1	−0.3 (2)	C7—C8—C9—C10	179.06 (15)
C1—C2—C3—C4	0.2 (2)	C13—C8—C9—Cl1	−178.03 (12)
N3—C2—C3—C4	178.97 (14)	C7—C8—C9—Cl1	0.8 (2)
C2—C3—C4—C5	−0.2 (3)	C8—C9—C10—C11	0.0 (3)
C2—C3—C4—N4	−179.79 (15)	Cl1—C9—C10—C11	178.32 (14)
O3—N4—C4—C3	0.1 (3)	C9—C10—C11—C12	−0.1 (3)
O4—N4—C4—C3	179.51 (17)	C10—C11—C12—C13	−0.1 (3)
O3—N4—C4—C5	−179.43 (18)	C11—C12—C13—C8	0.3 (3)
O4—N4—C4—C5	−0.1 (3)	C9—C8—C13—C12	−0.4 (2)
C3—C4—C5—C6	−0.1 (3)	C7—C8—C13—C12	−179.31 (15)
N4—C4—C5—C6	179.48 (16)		

### Hydrogen-bond geometry (Å, °)

**Table:**

*D*—H···*A*	*D*—H	H···*A*	*D*···*A*	*D*—H···*A*
N2—H1N1···O1	0.85 (2)	1.97 (2)	2.6081 (19)	131.2 (17)
C6—H6A···O3^i^	0.93	2.52	3.251 (2)	135

Symmetry codes:  (i) *x*, −*y*+1, *z*−1/2.
